# Quantifying the Search Behaviour of Different Demographics Using Google Correlate

**DOI:** 10.1371/journal.pone.0149025

**Published:** 2016-02-24

**Authors:** Adrian Letchford, Tobias Preis, Helen Susannah Moat

**Affiliations:** Data Science Lab, Behavioural Science, Warwick Business School, University of Warwick, CV4 7AL, Coventry, United Kingdom; University of Maribor, SLOVENIA

## Abstract

Vast records of our everyday interests and concerns are being generated by our frequent interactions with the Internet. Here, we investigate how the searches of *Google* users vary across U.S. states with different birth rates and infant mortality rates. We find that users in states with higher birth rates search for more information about pregnancy, while those in states with lower birth rates search for more information about cats. Similarly, we find that users in states with higher infant mortality rates search for more information about credit, loans and diseases. Our results provide evidence that Internet search data could offer new insight into the concerns of different demographics.

## Introduction

Our everyday interactions with large technological systems are generating records of human behaviour on a colossal scale [1–9]. Data drawn from mobile phone calls [10], public transport smart cards [11], financial markets [12–17], usage of Internet services [18–29], and even immense digitised collections of books [30–33] are being exploited to gather new insights into human health [34–36], mobility [10, 37], economic decision making [23, 38, 39] and more.

Here, we focus on search queries submitted to the Internet search engine *Google*. *Google* makes aggregated data on what people search for online available via its service *Google Trends*, offering unprecedented insight into people’s interests and concerns [4]. A number of studies have provided evidence that changes in the frequency with which *Google* users search for given terms across time not only correlate with changes in certain real world variables, such as unemployment rates, but may offer measurements of this behaviour before official data are released [40–45]. Further investigations have suggested that data on online information gathering may even anticipate future values of certain economic and behavioural indicators, such as box office movie revenue and financial market movements [38, 39, 46].

In this paper, we investigate whether or not we can identify a difference in online searches between people in different demographics. Instead of using data drawn from *Google Trends*, we use a service called *Google Correlate* [47, 48]. This service allows a user to input either a time series or data relating to U.S. states, and returns the search terms for which the number of searches is most strongly correlated across time or across states.

However, the correlation coefficients which are returned by *Google Correlate* need to be treated with care. Firstly, the system reports only the highest correlations out of potentially hundreds of millions which greatly increases the chances of finding spurious correlations. Secondly, the search data retrieved from neighbouring states may not be independent and the distribution of search volume may not be Gaussian, such that the data break the assumption of traditional correlation coefficient tests [49]. Thirdly, *Google Correlate* uses a hashing algorithm to improve the speed of searching millions of time series [48]. However, this applies to time series analysis only. Furthermore, *Google* restricts access to their full dataset hindering development of specialised statistical tests.

As a case study, we use *Google Correlate* to investigate how the searches of *Google* users vary across U.S. states with different birth rates and infant mortality rates. We seek to determine which correlations are significant among potentially hundreds of millions of correlations when the data do not follow traditional assumptions. To investigate the results from the case study, we develop a bootstrap statistical test.

Many different demographic variables are measured across US states. Further studies could use the approach we present to extend this investigation to other demographic variables.

## Case study

**Fig 1:**
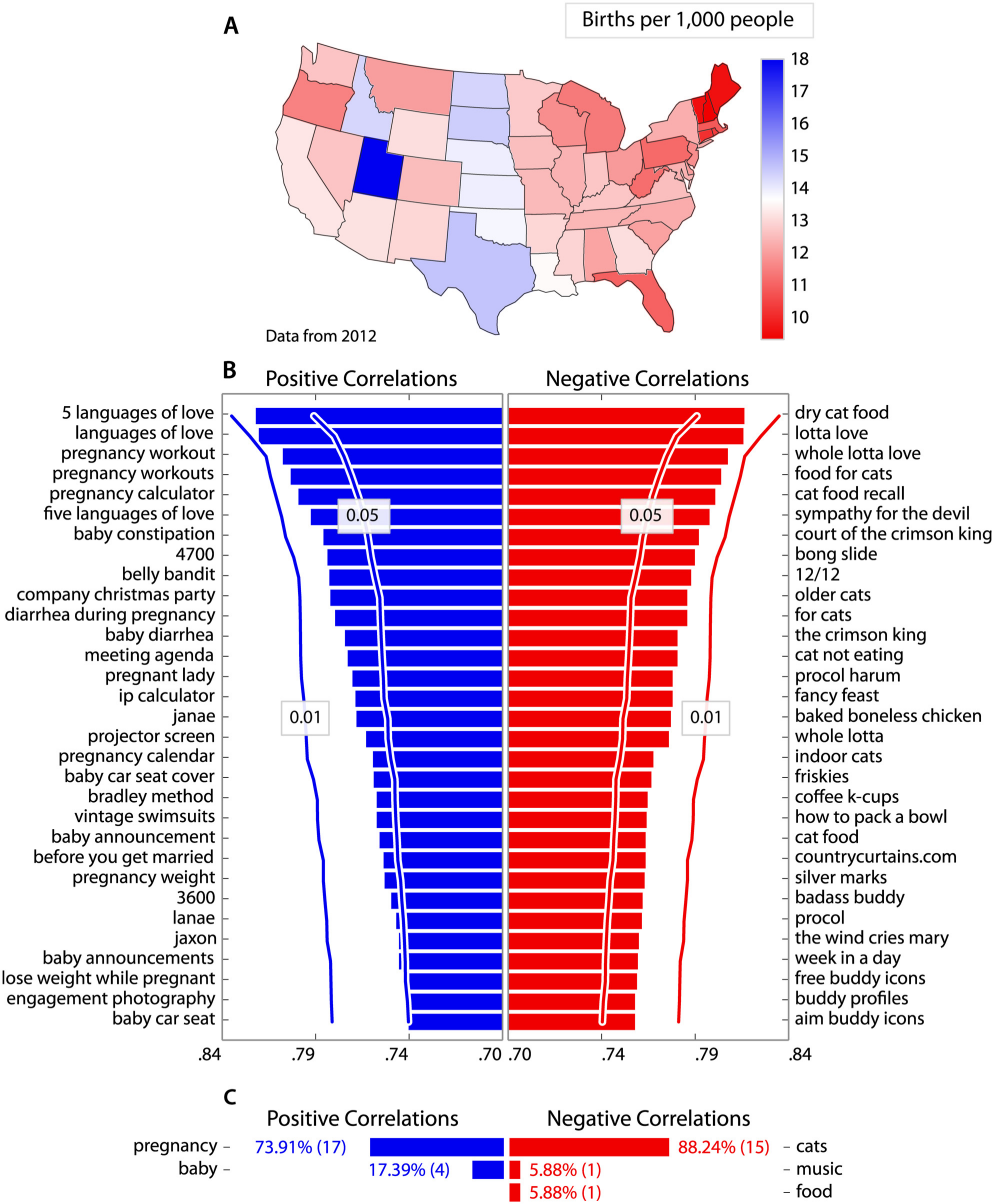
How do *Google* queries vary with birth rate? (**A**) The number of births for 1,000 people in each US state. Birth rate is defined as the number of births for 1,000 people. (**B**) We use *Google Correlate* to find terms for which the number of searches is higher in U.S. states with higher birth rates. Similarly, we identify terms for which the number of searches is higher in states with lower birth rates. Here, we list the 31 terms which showed the strongest positive correlation (left) and negative correlation (right) with state wide birth rate. To determine the significance of these correlations, we generate 1,000 random samples from a multivariate Gaussian distribution where states which are closer together tend to have a similar value. We submit these samples to *Google Correlate* and build a distribution of correlation coefficients for each of the 31 top most search terms. We depict the strength of correlation required for the correlation to be significant at the *p* < 0.05 and *p* < 0.01 level, given this null hypothesis distribution. (**C**) To allow us to generalise beyond individual search terms, we conduct an online survey asking participants to identify the main topic in each list of 31 terms. Here, we depict all survey responses which account for more than 5% of submitted responses. Our results suggest that users in states with higher birth rates search for more information about pregnancy, while those in states with lower birth rates search for more information about cats (“baby car seat”, *p* = 0.051, all remaining *p*s <0.05).

We retrieve the number of births per 1,000 people in each U.S. state in 2012 from the *Centers for Disease Control and Prevention* on 27 May 2014 (http://wonder.cdc.gov/natality.html) (Fig 1A).

We retrieved the list of search terms for which search volume was most strongly positively correlated with birth rate by state by submitting the birth rate data to *Google Correlate* (http://www.google.com/trends/correlate) on 27 May 2014. On the left hand side of Fig 1B, we list the 31 terms for which search volume exhibits the strongest positive correlation with birth rate for a state. We retrieve the list of negatively correlated terms by multiplying the birth rate for each state by −1, before submission to *Google Correlate*. We list the 31 terms for which search volume exhibits the strongest negative correlation with birth rate for a state on the right hand side of Fig 1B.

We observe that particular topics emerge within the lists of terms that *Google Correlate* returns. For example, search terms for which searches are higher in states with higher birth rates include “pregnancy workout”, “baby constipation” and “baby announcement”. Search terms for which searches are higher in states with lower birth rates include “dry cat food”, “older cats” and “cat not eating”.

To allow us to generalise beyond single keywords and interpret these datasets in an objective fashion, we conduct an online survey using *Amazon Mechanical Turk*. *Amazon Mechanical Turk* is a service which allows users to post tasks that they wish other users to complete, in exchange for a small fee. For each list of 31 terms, we ask participants, “What is the most prominent topic in these phrases?” Responses are limited to one word, and each participant is only allowed to respond once to each question. In total, we analyse 40 responses received from *Amazon Mechanical Turk* users, with 23 responses for the positively correlated terms, and 17 responses for the negatively correlated terms. Details of the survey can be found in the *Supporting Information*.

In Fig 1C, we depict all survey responses which account for more than 5% of submitted responses, along with the percentage and number of respondents who gave each response. We find that 74% of respondents judge that the search terms for which the number of searches is higher in states with higher birth rates relate to “pregnancy”. Conversely, we find that 88% of respondents judge that the search terms for which the number of searches is higher in states with lower birth rates relate to “cats”. We investigate the statistical significance of these correlations in the following section.

**Fig 2:**
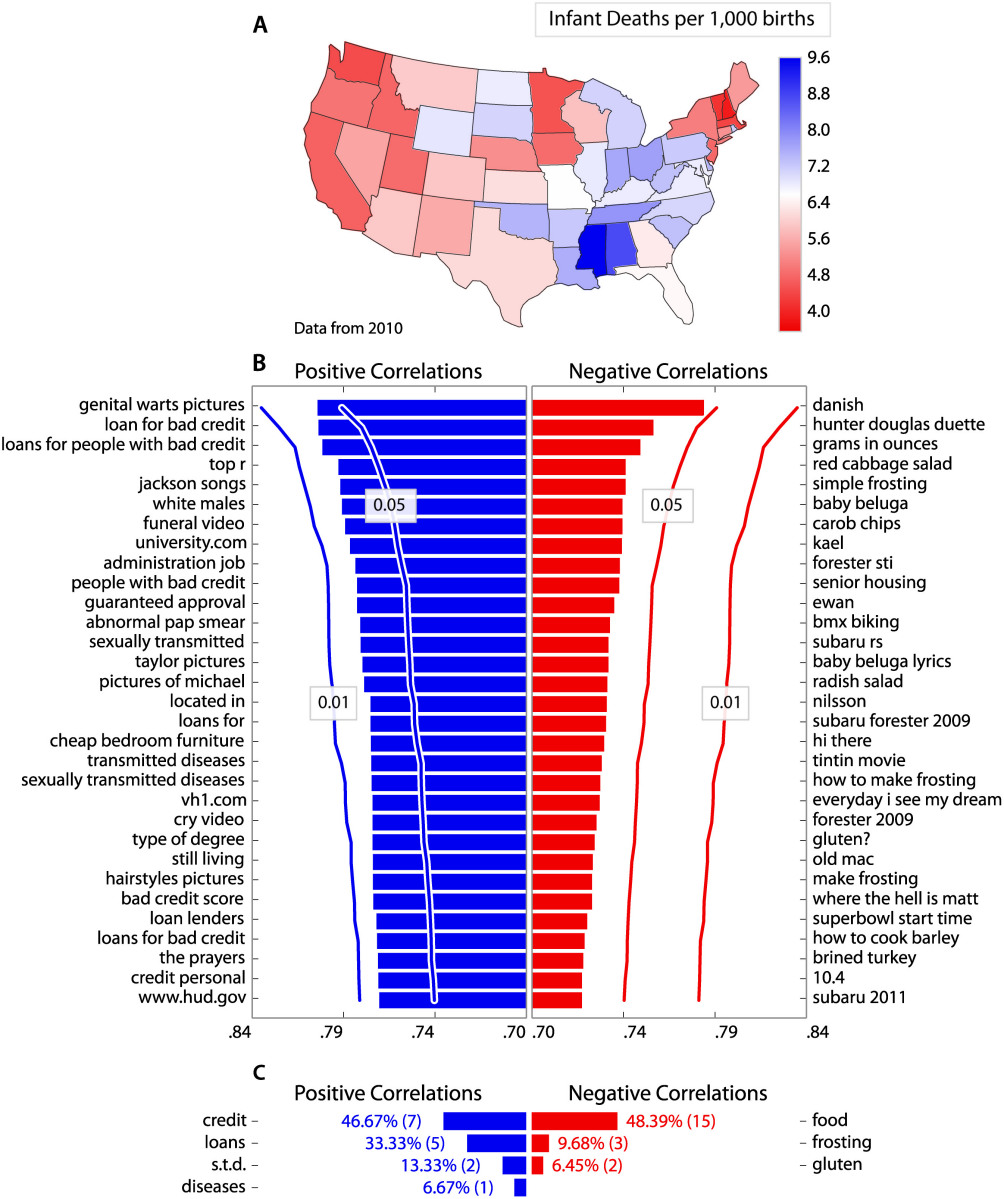
How do *Google* queries vary with infant mortality rate? (**A**) Infant mortality rates for each state in the US. An infant is defined as any person one year old or younger. Infant mortality rate is defined as the number of infant deaths per 1,000 births. (**B**) In a similar fashion to our investigation of birth rates (Fig 1), we use *Google Correlate* to find terms for which the number of searches is higher in U.S. states with higher infant mortality rates, and with lower infant mortality rates. We list the 31 terms for which differences in search volume across U.S. states shows the strongest positive correlation (left) and negative correlation (right) with state wide infant mortality rate. Again, we generate 1,000 random samples from a multivariate Gaussian distribution where states which are closer together tend to have a similar value. We submit these samples to *Google Correlate* and build a distribution of correlation coefficients for each of the 31 top most search terms. We depict the strength of correlation required for the correlation to be significant at the *p* < 0.05 and *p* < 0.01 level, given this null hypothesis distribution. (**C**) Again, we ask *Amazon Mechanical Turk* users to identify the most prominent topic in each of these lists of terms. We depict all survey responses which account for more than 5% of submitted responses, along with the percentage and number of respondents who gave each response. Our results suggest that users in states with higher infant mortality rates search for more information about credit and loans, as well as sexually transmitted diseases (all search terms *p* < 0.05).

We repeat this process using data on the number of infant deaths per 1,000 births for each state in 2010 downloaded from the *Centers for Disease Control and Prevention* on 27 May 2014 (http://wonder.cdc.gov/lbd.html) (Fig 2A). An infant is defined as any person one year old or younger. In Fig 2B, we list the 31 terms for which search volume exhibits the strongest positive correlation with infant mortality rate for a state (left), and the strongest negative correlation with infant mortality rate for a state (right).

Again, we note that certain topics are apparent within these lists. For example, search terms for which searches are higher in states with higher infant mortality rates include “loan for bad credit” and “people with bad credit”, as well as “abnormal pap smear” and “transmitted diseases”. Search terms for which searches are higher in states with lower birth rates include “red cabbage salad”, “simple frosting” and “carob chips”.

Once more, we ask *Amazon Mechanical Turk* users to identify the most prominent topic in each of these lists of terms. In total, we analyse 46 responses received from *Amazon Mechanical Turk* users, with 15 responses for the positively correlated terms, and 31 responses for the negatively correlated terms.

In Fig 2C, we depict all survey responses which account for more than 5% of submitted responses, along with the percentage and number of respondents who gave each response. We find that 80% of respondents judge that the search terms for which the number of searches is higher in states with higher infant mortality rates relate to “credit” or “loans”, and 20% of respondents judge that these terms relate to “s.t.d” or “diseases”. Conversely, we find that 48% of respondents judge that the search terms for which the number of searches is higher in states with lower infant mortality rates relate to “food”, with 10% of users suggesting “frosting”, and 6% suggesting “gluten”.

## Methods

We construct a method to test whether the strength of the correlations for the most correlated search terms for birth rates and infant mortality rates is statistically significant. We note that in Fig 1A the birth rates are not independently distributed. Visual inspection indicates that states which are closer together tend to have similar birth rates. The traditional statistical test for Pearson’s correlation coefficient explicitly requires the observations to be independent [49]. To overcome this problem, we perform a bootstrapped statistical test of the correlation between the birth rates and search data. We assume that the birth rates and infant mortality rates are drawn from a multivariate Gaussian distribution. We generate random samples from this distribution and submit each one to *Google Correlate* and build a distribution of the highest correlation coefficients returned by this system.

We set the multivariate Gaussian distribution with a mean of zero and a covariance matrix **K** which accounts for potential covariance between US states. Denoting the geographic data as a vector **y**, we write our model as:

y∼N(0,K) (1)

In our model, two states are more dependent on each other if they are physically closer together. We imagine that the states are like vertices in a graph and the edges represent shared borders. The distance between any two states is the length of the shortest path between them. For example, California and Nevada have a distance of 1 because they share a border, that is, they are connected on the network. California and Utah have a distance of 2 because the shortest path between them is two edges long. Each state’s distance from itself is zero. The distance between all US states and Alaska and Hawaii is set to ∞.

We specify a distance matrix, **D**, where each cell is the squared distance between two states. We then write the covariance matrix as a Gaussian function of **D**:

$$K=b\cdot e^{-aD}+cI$$ (2)

We select the parameters *a*, *b* and *c* by maximising the likelihood function of both the birth rates and infant mortality rates. The log likelihood function of the parameters given Eq (1) is:

$$\log p\left(y|a,b,c\right)=-\frac{1}{2}y^{\text{T}}K^{-1}y-\frac{1}{2}\log\left|K\right|-\frac{n}{2}\log2\pi$$ (3)

and the log likelihood of the parameters given both the birth rates and infant mortality rates is:

$$\log p\left(y_{b}|a,b,c\right)+\log p\left(y_{m}|a,b,c\right)$$ (4)

where **y**_*b*_ represents the birth rates and **y**_*m*_ represents the infant mortality rates. We use the downhill simplex algorithm [50] to maximise Eq (4). We run the algorithm 10 times with the starting values for *a*, *b*, and *c* drawn from a standard Gaussian distribution and select the result which maximises the log likelihood. The values we find are *a* = 0.0816628, *b* = 0.75687803, *c* = 0.44543885.

Previous studies have used other methods of quantifying the relationship between geographic regions. For example, the spread of epidemics can be modelled with the air traffic connecting global regions [51, 52]. Future analysis could investigate whether a different metric of distance between states would improved the fit of the multivariate Gaussian to the demographic data.

We generate 1,000 random samples from this multivariate Gaussian distribution and submit each one to *Google Correlate*. For each sample, *Google Correlate* returns a maximum of 100 terms for which search volume is most correlated with the sample and where the Pearson’s correlation coefficient is equal to or above 0.6. The left panel of Figure C in S1 Text depicts the distribution of Pearson’s correlations for the random samples. We compile the cumulative distribution function (CDF) of the correlation coefficient for each *k*^th^ most correlated search term. These distributions represent the distribution of the correlation coefficient we would expect under the null hypothesis that the submitted dataset is drawn from a multivariate Gaussian distribution with mean 0 and covariance **K**, with no relationship to the *k*^th^ search term. The right panel of Figure C in S1 Text shows the CDF of the first (*k* = 1) search term. We use these distributions to statistically test the correlation coefficient of the search volume of each search term with both the birth rate and infant mortality rate data.

## Results

We find that all search terms that *Google Correlate* lists as both positively and negatively correlated with birth rates are statistically significant at the *p* < 0.05 level. Only the least most positively correlated term, “baby car seat”, is not significant at the *p* < 0.05 level. All terms that are most positively correlated with infant death rates are all significant at the *p* < 0.05 level. The most negatively correlated terms with infant death rates are not significantly correlated.

## Discussion

In this study, we investigate how searches of *Google* users vary across U.S. states with different birth rates, by using the service *Google Correlate*. We find that as the number of babies born per 1,000 inhabitants increases, the number of searches for information about pregnancy also increases, as one might expect. However, as birth rate decreases, our analysis reveals increases in the number of searches about cats.

In a second analysis, we consider differences in search activity in states with different infant mortality rates. We find that as the proportion of babies who do not live until the age of one increases, the number of searches for information about credit, loans and sexually transmitted diseases also increases.

Previous studies have demonstrated how data on *Google* usage retrieved from the *Google Trends* interface can reveal interesting relationships between online behaviour and various measures of behaviour in the real world, such as reports of infections of influenza like illnesses [44, 53], fluctuations in stock markets [38, 39, 54, 55], measures of risk of investment [14, 56] and unemployment claims [42].

However, search volume data can only be retrieved from *Google Trends* if the user specifies the search terms of interest. Researchers interested in the link between *Google* usage and real world behaviour may select a set of terms which they believe to be related to the behaviour of interest, or generate lists of terms which cover a range of different topics [38]. It is not possible to submit data relating to real world behaviour and automatically retrieve search terms where search behaviour reflects the submitted real world data.

Comparison of search behaviour across geographic areas is also challenging when using the *Google Trends* interface. When data is requested for a specific geographic area, *Google Trends* scales the maximum value in the retrieved data to 100. For this reason, data retrieved for multiple geographic areas cannot be directly compared, unless data for two keywords is retrieved simultaneously and the ratio between these two keywords is calculated [29, 57].

The *Google Correlate* service offers a solution to both of these problems. Users are able to input data which varies across time or across US states, and retrieve search terms for which the frequency of searches is most correlated with the input data. The interface also returns the strength of correlation for each of these search terms. However, no method has been proposed to determine whether correlations of the observed strength might be expected simply as a result of *Google Correlate* evaluating search volume data for an extremely large number of search terms.

In this paper, we demonstrate how *Google Correlate* can be used to identify the search terms for which search activity is most correlated with the real world data provided—for example, per state birth rates or infant mortality rates. Crucially, we develop a statistical test to determine how likely it would be to observe correlations of this strength under a null hypothesis of no relationship between the search term and the real world data. According to this method, all but one of the terms for which search volume is most positively and negatively correlated with birth rates are significantly correlated at the *p* < 0.05 level. The terms for which search volume is most positively correlated with infant mortality rates are significant at the 0.05% level. However, we find no evidence that the strength of the correlations for the terms most negatively correlated with infant mortality rates is significant.

We highlight that the presence of relationships at the aggregate level does not imply the presence of similar relationships at the individual level. For example, our finding that Internet users in states with lower birth rates search for more information about cats does not allow us to conclude that individuals with lower birth rates search for more information about cats. Furthermore, while our statistical method allows us to demonstrate a significant correlation between interest in certain search terms and demographics, our analysis does not imply causation. For example, poor education and low wages might be a factor that causes a decrease in infant survival rates as well as interest in credit, loans and sexually transmitted diseases.

In this paper, we propose a method to statistically evaluate search behaviour data provided by *Google Correlate*. The results of our two case studies suggest that appropriate analyses of Internet search data could offer new insights into the concerns of different demographics. Combined with data on real world economic and health variables, search engine data may allow us to gain a better understanding of the different worlds experienced by different sectors of society.

## Supporting Information

S1 Text Contains details on the *Amazon Mechanical Turk* survey and the bootstrapped statistical test. This document describes a survey conducted on the online *Amazon Mechanical Turk* system. It includes the raw responses and the methods we used to clean the data. We also include in this document figures of the distribution of Pearson’s correlation as returned by *Google Correlate*.
(PDF)

S1 Data The data. Contains all results from *Google Correlate* used in this study.
(TXT)
