# Supplementary material for: Quantifying the Search Behaviour of Different Demographics Using Google Correlate
Source: PLoS One. 2016 Feb 24;11(2):e0149025. doi: 10.1371/journal.pone.0149025 (PMC4766235; doi:10.1371/journal.pone.0149025)
Supplement: S1 Text — This document describes a survey conducted on the online Amazon Mechanical Turk system. It includes the raw responses and the methods we used to clean the data. We also include in this document figures of the distribution of Pearson’s correlation as returned by Google Correlate. (PDF) [file pone.0149025.s001.pdf]

Quantifying the search behaviour of different demographics using *Google Correlate*

Supporting Information

Adrian Letchford*, Tobias Preis, and Helen Susannah Moat

Data Science Lab, Behavioural Science, Warwick Business School, University of Warwick, CV4 7AL, Coventry, UK

* To whom correspondence should be addressed; E-mail: Adrian.Letchford@wbs.ac.uk

February 15, 2016

Allocating topic names to lists of search terms using *Amazon Mechanical Turk*

To allocate topic names to the lists of search terms, we conduct an online survey by creating an *Amazon Mechanical Turk* (`http://www.mturk.com`) task, known as a "Human Intelligence Task" (HIT).

Participants were presented with search terms from one of the four lists retrieved from *Google Correlate*, and asked, "What is the most prominent topic in these phrases?" To make our task feasible for users, we restricted these lists to the most strongly correlated 31 terms, rather than the full 100 returned by *Google Correlate*. The four lists of search terms presented to participants are depicted in Figs. 1B and 2B of the main manuscript. These lists consisted of terms for which search volume for a U.S. state was most strongly positively correlated with birth rate, negatively correlated with birth rate, positively correlated with infant mortality rate, or negatively correlated with infant mortality rate.

We recruited *Mechanical Turk* users who had previously had their responses to 5000 HITs approved by the task creator, and who had an approval rate of at least 98% for all previously completed HITs. Participants were paid 0.10 USD per response. Responses were limited to one word, and each participant was only allowed to respond once to each question. The raw survey responses are listed in Figs. A and B.

The survey consisted of two steps, as detailed below.

Survey step 1

Participants were presented with instructions as reproduced below.

Instructions

Please follow these instructions when answering this survey:

- Read the question carefully.
- Write your answer with ONLY one word. We do not accept answers of more than one word.

Survey step 2

Participants were then presented with the following question: "What is the most prominent topic in these phrases (one word only)?" Underneath the question, we displayed one of the four lists of strongly correlated words retrieved from *Google Correlate*.

Data cleaning

Before analysis, all responses were converted to lower case. Singular and plural forms of the same word (e.g., "cat" and "cats") and responses which only differed in punctuation (e.g., "s.t.d." and "std") were grouped together.

Raw survey responses

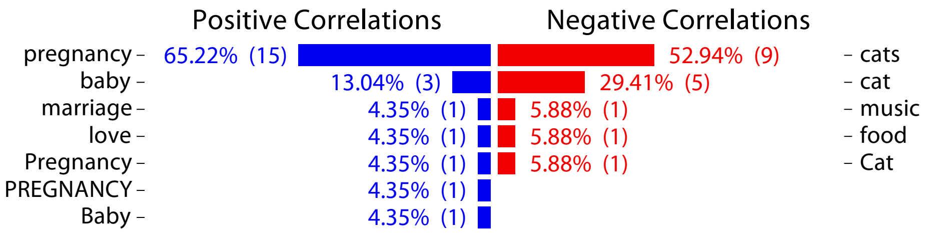

Figure A: Topic labels provided for search terms correlated with state birth rate. Here, we depict the raw survey responses, along with the percentage and number of respondents who gave each response. The converted and grouped terms are shown in Fig. 1C of the main manuscript.

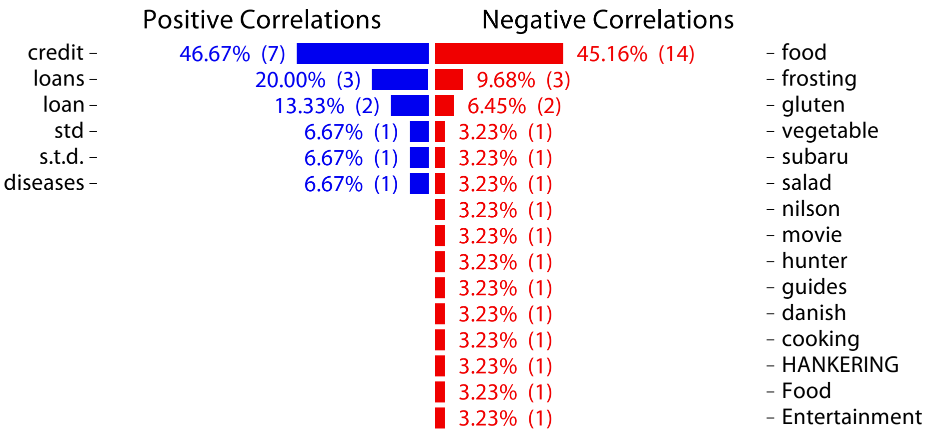

Figure B: Topic labels provided for search terms correlated with state infant mortality rate. As in Fig. A, we depict the raw survey responses, along with the percentage and number of respondents who gave each response. The converted and grouped terms are shown in Fig. 2C of the main manuscript.

Bootstrapped distributions of Google Correlate results

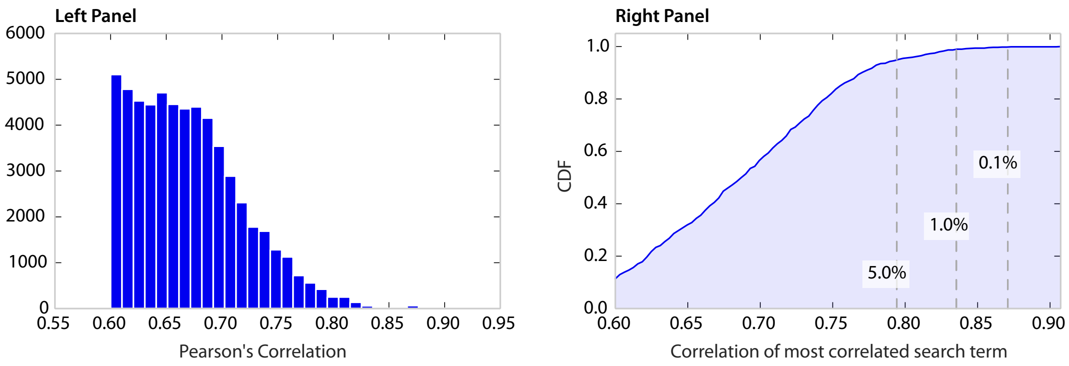

Figure C: Statistically testing Google Correlate's results. We note in Fig. 1A of the main manuscript that birth rates are not independently distributed. States that are closer together have a similar birth rate. The traditional statistical test for significant correlations requires observations to be independent. To overcome this problem, we use a multivariate Gaussian distribution to model the dependence between states. We generate 1,000 random samples from this distribution submitting each one to *Google Correlate*. (**Left Panel**) Here we depict the distribution of the highest correlation coefficients for each random sample. *Google Correlate* does not return any search terms for which the correlation coefficient is below 0.6. (**Right Panel**) We plot the cumulative distribution function of the highest correlation coefficient for each random sample. Here, we identify the correlation coefficient for which the probability of sampling a correlation coefficient of the same or greater strength from this distribution is 5%, 1% and 0.1%. This distribution represents the null hypothesis that the submitted dataset is drawn from a multivariate Gaussian distribution and has no relationship to the search volume for the most correlated search term.
